# Radiofrequency Ablation of Re-entrant Atrial Tachycardia Originating from the Superior Vena Cava with a High-resolution, 3-dimensional Mapping System

**DOI:** 10.19102/icrm.2023.14083

**Published:** 2023-08-15

**Authors:** Kiichi Miyamae, Yosuke Murase, Yasuhiro Ogawa, Hajime Imai, Naoaki Kano, Keita Mamiya, Tomoyo Ikeda, Shinji Yamazoe, Jun Torii, Kazuyuki Yamanaka, Katsuhiro Kawaguchi

**Affiliations:** ^1^Department of Cardiology, Komaki City Hospital, Komaki, Japan

**Keywords:** Atrial tachycardia, entrainment pacing, high-resolution 3D mapping, re-entry, superior vena cava

## Abstract

An 80-year-old man with no previous history of catheter ablation or cardiac surgery underwent catheter ablation for atrial tachycardia (AT). We suspected that the mechanism causing AT was re-entry indicated by the entrainment phenomenon during AT and through activation mapping with a 3-dimensional mapping system (EnSite™ X EP system; Abbott, Chicago, IL, USA). We used a multipolar catheter (Advisor™ HD Grid Mapping Catheter; Abbott) inserted into the superior vena cava (SVC) to accomplish activation mapping. The AT circuit was localized inside the SVC with a fractionated potential recorded on its right lateral wall. A similar fractionated potential was observed in the surrounding area. These areas functioned as the critical isthmus of the AT. Radiofrequency (RF) catheter ablation at these sites eliminated the tachycardia. After RF delivery, no tachycardia was induced by programmed stimulation, even during isoproterenol infusion. Consequently, there was no recurrence of tachycardia even after catheter ablation.

## Introduction

Myocardial fibers arising from the right atrium (RA) and extending to the superior vena cava (SVC) have been demonstrated to exist through histologic and physiologic studies.^1,2^ These myocardial fibers have previously been shown to cause atrial fibrillation (AF) and other atrial arrhythmias, and they are known therapeutic targets for catheter ablation.^3^ In this case report, we successfully demonstrated and treated a re-entrant atrial tachycardia (AT) with slow conduction in the SVC through electrophysiological means and using a high-resolution 3-dimensional (3D) mapping system.

## Case presentation

**Figure 1:**
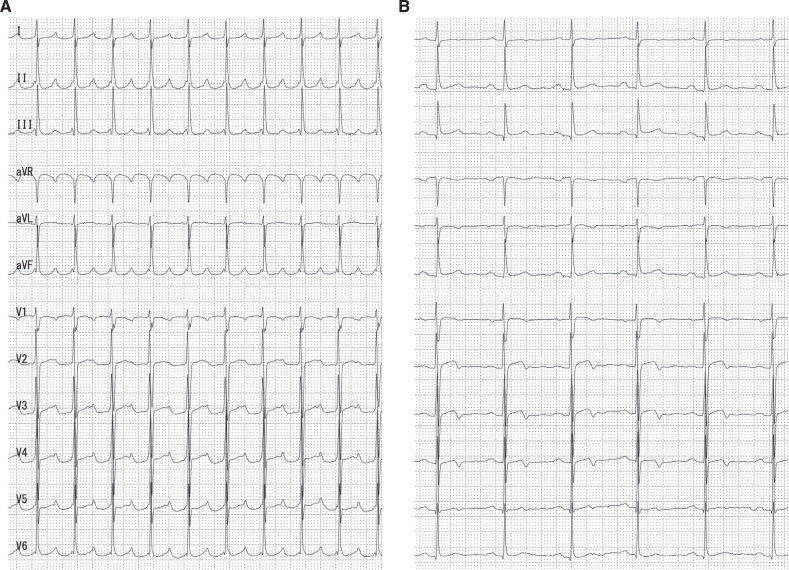
**A:** A 12-lead electrocardiogram showing atrial tachycardia at a P rate of 240 beats/min with 2:1 atrioventricular conduction. **B:** A 12-lead electrocardiogram showing sinus rhythm with a normal P-wave morphology.

The patient was an 80-year-old man who had been aware of palpitations and shortness of breath with light exertion for several years. The patient was consequently referred to our hospital by his family physician for the treatment of AT. He did not have any prior history of cardiac surgery or catheter ablation. A 12-lead electrocardiogram (ECG) of AT showed a P–P interval of 240 beats/min with 2:1 atrioventricular conduction. The P-wave morphology at AT was positive in leads I, II, III, and aVF and biphasic in V1. The P-wave morphology was similar to that during sinus rhythm, but the height of the P-waves in leads II, III, and aVF was taller than that seen in sinus rhythm **(Figure 1)**. No structural abnormalities were visualized by echocardiography.

### Electrophysiologic study and radiofrequency ablation

**Figure 2:**
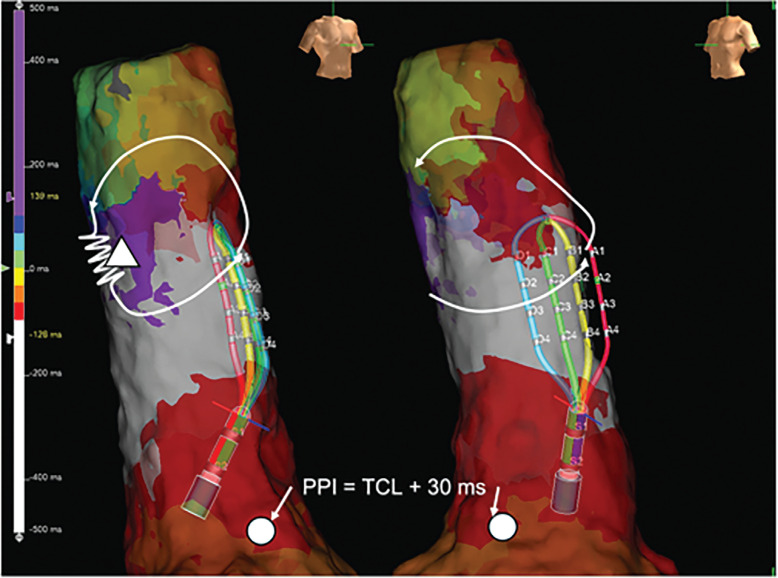
A local activation time map of the superior vena cava and right atrium during atrial tachycardia in the right anterior oblique 30° and left anterior oblique 45° views. The white circles indicate areas where the post-pacing interval was measured. *Abbreviations:* PPI, post-pacing interval; TCL, tachycardia cycle length.

**Figure 3:**
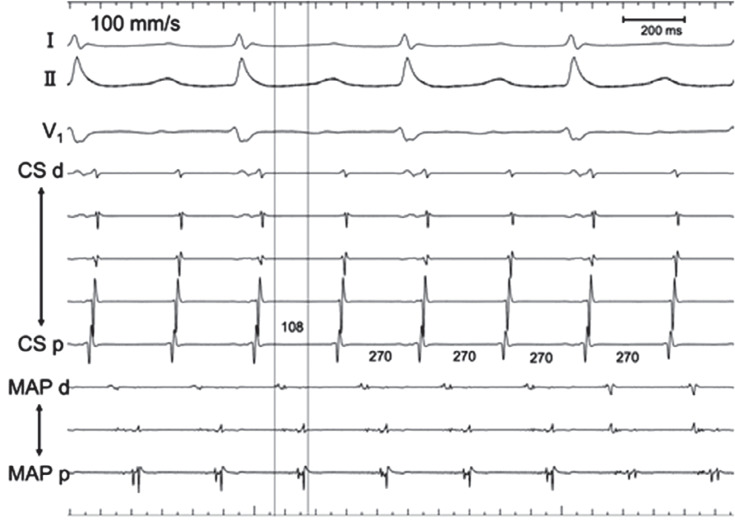
A surface electrocardiogram and intracardiac electrograms were recorded during atrial tachycardia. The cycle length of the tachycardia was 270 ms. The mapping catheter (MAP) was located at the isthmus of the tachycardia, as shown in **Figure 2** (indicated by a white triangle). The fragmented potentials were recorded at MAP.

**Figure 4:**
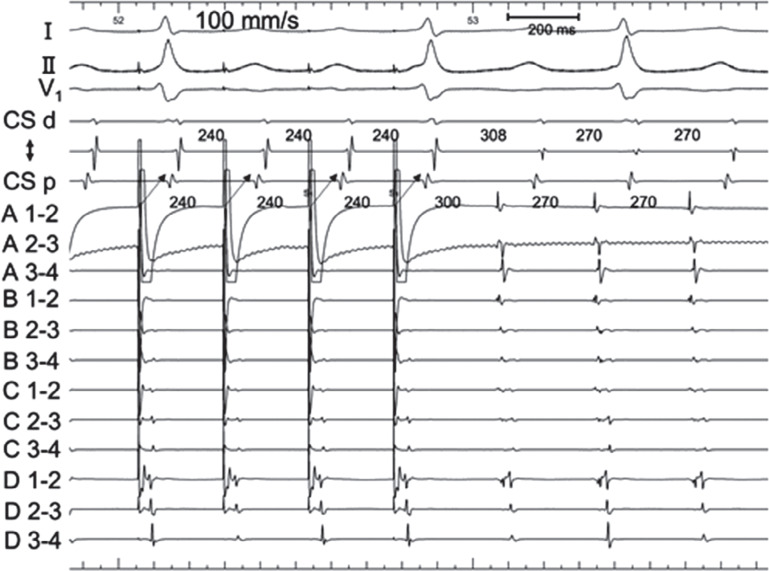
Entrainment pacing was performed at the junction between the right atrium and superior vena cava, which was located out of the tachycardia circuit (lower circle in **Figure 2**). The post-pacing interval was 300 ms and the tachycardia cycle length was +30 ms, indicating the site was located away from the tachycardia circuit. Entrainment pacing from this site showed that the coronary sinus potential was captured antidromically (arrow).

**Figure 5:**
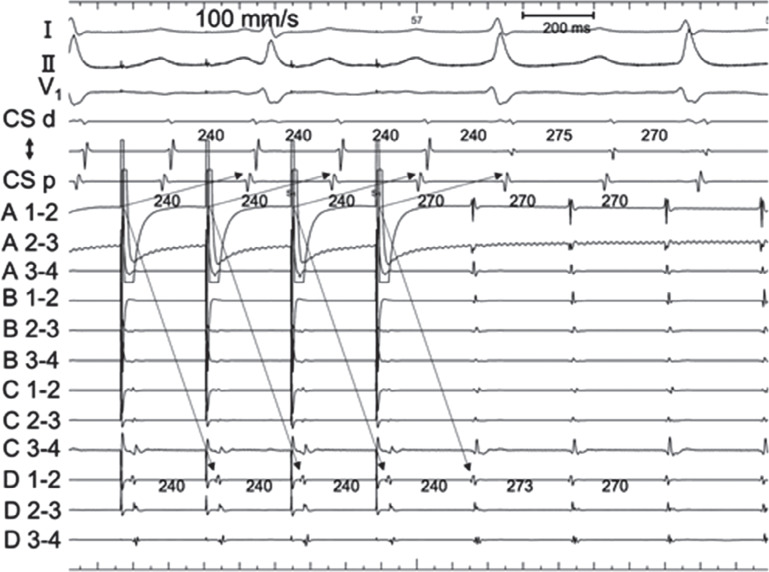
Entrainment pacing was performed from A1–2 of the HD Grid placed at the site on the tachycardia circuit (as **Figure 2**). This tracing exhibits manifest entrainment, with a post-pacing interval identical to the tachycardia cycle length, proving that the mechanism is re-entry. The coronary sinus potential and spline D were orthodromically captured (arrows).

**Figure 6:**
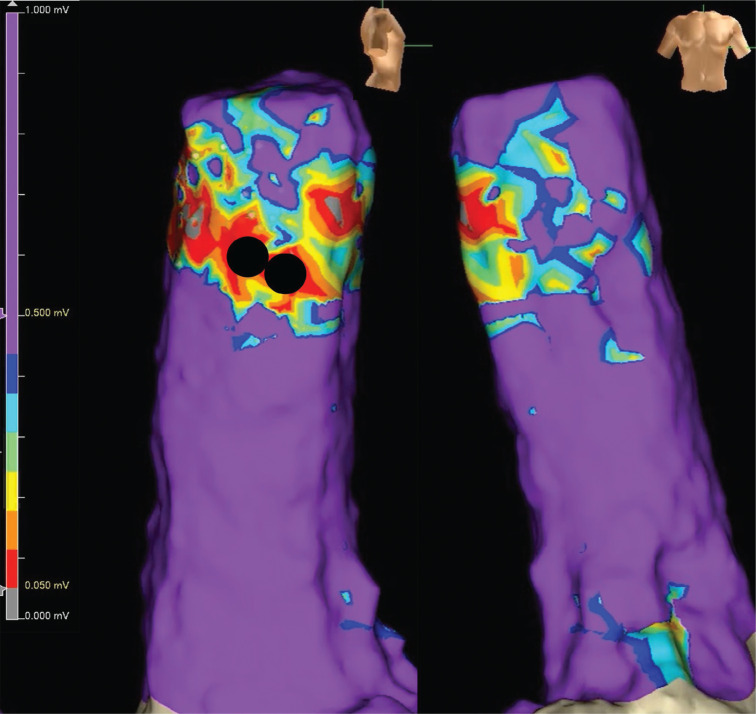
The voltage map during sinus rhythm showed that we succeeded in treating the atrial tachycardia without superior vena cava isolation. The circles indicate the areas where ablation was performed. The low-voltage cutoff was 0.005 mV. **A:** Right-sided view. **B:** Frontal view.

An electrophysiologic study was performed on the patient in the fasting state without sedation. Intra-atrial mapping was implemented using a 6-French (Fr) 10-pole electrode catheter positioned inside the lateral side of the RA and through a 6-Fr 10-pole electrode catheter inserted into the coronary sinus (CS). The cycle length of AT was 270 ms, with the earliest atrial activation occurring at the high lateral RA. On entrainment pacing at the lower lateral RA, the proximal CS, and the distal CS, the post-pacing interval (PPI) was prolonged compared to the tachycardia cycle length (TCL) (lower lateral RA, 402 ms; proximal CS, 406 ms; distal CS, 464 ms). We conducted local activation time (LAT) mapping using a 3D mapping system (EnSite™ X EP system; Abbott, Chicago, IL, USA) and a multipolar catheter (Advisor™ HD Grid Mapping Catheter; Abbott). The LAT map of the AT localized the tachycardia circuit to the SVC **(Figure 2 and Video 1)**. A slow conduction area appeared to be the critical isthmus of the AT observed in the right lateral SVC wall. Fragmented potentials were observed at the multipolar catheter located at the slow conduction area, about 4.1 cm above the SVC–RA junction. The potentials were 108 ms ahead of the P-wave of the surface ECG **(Figure 3)**. We performed entrainment pacing with the multipolar catheter positioned at the SVC–RA junction and at the SVC anterior wall that appeared to be on the AT circuit. The PPIs were 300 and 270 ms, respectively **(Figures 4 and 5)**. Pacing with A1–2 of the HD Grid exhibited manifest entrainment, with a PPI identical to the TCL, proving that the mechanism was re-entry. Splines B and C of the HD Grid were antidromically captured, whereas spline D was orthodromically captured; this indicated that the pacing site was located upstream of the slow conduction zone. We decided to ablate what we considered to be the critical isthmus of this AT. We used a contact force-sensing irrigated radiofrequency (RF) ablation catheter (TactiCath; Abbott). RF energy was delivered at 50°C and 30 W for 60 s to ablate the critical isthmus and the affected areas around it. The tachycardia was terminated within 2.8 s with a prolonged TCL. After RF delivery, tachycardia was no longer induced by programmed stimulation, even during isoproterenol infusion. No complications, such as sick sinus syndrome or phrenic nerve paralysis, were noted. We obtained another activation map during sinus rhythm **(Figure 6)**, which showed that we had succeeded in treating AT without SVC isolation.

## Discussion

The present report demonstrated effective management of AT originating from the SVC with catheter ablation. LAT mapping using a high-resolution 3D mapping system revealed the AT circuit very clearly. The earliest fractionated potential preceded the P-wave of the surface ECG at the right lateral SVC; RF applied to this site eliminated the AT. This long, fractionated potential was considered to be the critical isthmus potential of the AT circuit.

The present case recorded SVC potentials around the SVC–RA junction and the myocardial sleeve inside the SVC. The distance measured with the 3D mapping system in this case (4–6 cm) was slightly longer than that in previous studies.^4^ These myocardial sleeves are known to be the substrate of atrial arrhythmias, including AF, and are the main target of catheter ablation.^3,5^ Several cases of AT originating from the SVC have been reported. Chang et al. reported 3 cases of focal AT originating from various parts of the SVC.^5^ The ectopic foci of SVC shown in these cases were speculated to contribute to abnormal automaticity or triggered activity. On the other hand, there are some reports affirming that the mechanism of AT is re-entry.^6,7^ Shah et al. reported that the myocardial sleeve in the SVC has an anisotropic substrate, producing a patchy incomplete block and delayed activation via a basket catheter. They demonstrated that this electrophysiologic substrate is related to the re-entry mechanism.^6^ Okamoto et al. also reported that the mechanism of AT originating from the SVC was re-entry, having established this finding using the reset phenomenon and entrainment pacing during tachycardia.^7^ To the best of our knowledge, there are no reports of using high-resolution 3D mapping systems for mapping the circuit of localized re-entrant ATs confined to the SVC, which was instrumental in identifying the optimal ablation site.

The minor limitation of this case was that ablation sites were chosen based on a few points of slow conduction areas for AT without performing SVC isolation. This may cause the patient to develop other arrhythmias in the future, such as SVC-triggered AF. However, no atrial premature contractions of SVC origin occurred with isoproterenol infusion in our case. SVC isolation can only be accomplished with circumferential ablation, but this increases the risk of phrenic nerve paralysis, sick sinus syndrome, or SVC stenosis, so we did not perform this procedure in order to avoid these complications.

Our report demonstrated a re-entrant AT that rendered a slow conduction zone in the SVC as a critical isthmus. This was a rare case of re-entrant AT involving the SVC, with high-resolution 3D mapping serving to localize the tachycardia circuit to the SVC.

## Figures and Tables

**Video 1:** Sparkle map during atrial tachycardia of the superior vena cava and right atrium in the right anterior oblique 30° and left anterior oblique 45° views.
